# Volume of the proximal half of the thoracic aorta is the most comprehensive FDG-PET/CT indicator of arterial aging throughout adulthood

**DOI:** 10.1186/s41824-023-00169-2

**Published:** 2023-06-28

**Authors:** Moira Hurstel, Laure Joly, Laetitia Imbert, Gaetan Zimmermann, Véronique Roch, Pauline Schoepfer, Zohra Lamiral, Paolo Salvi, Athanase Benetos, Antoine Verger, Pierre-Yves Marie

**Affiliations:** 1 Department of Nuclear Medicine and Nancyclotep Molecular Imaging Platform, CHRU-Nancy, Université de Lorraine, 54000 Nancy, France; 2 Geriatric Department, CHRU Nancy, Université de Lorraine, Nancy, France; 3 INSERM, DCAC, Université de Lorraine, Vandœuvre-lès-Nancy, France; 4 IADI, INSERM U1254, Université de Lorraine, Nancy, France; 5 Department of Cardiology, Istituto Auxologico Italiano, IRCCS, Milan, Italy; 6 INSERM, CIC 1433, Université de Lorraine, CHRU-Nancy, Nancy, France; 7 Médecine Nucléaire, Hôpital de Brabois, CHRU-Nancy, rue Morvan, 54500 Vandoeuvre-lès-Nancy, France

**Keywords:** ^18^F-fluorodesoxyglucose, PET/CT hybrid imaging, Arterial aging, Aorta stiffness, Blood pressure

## Abstract

**Introduction:**

^18^F-fluorodeoxyglucose positron emission tomography (FDG-PET) and computed tomography (CT) features of the proximal and more elastic half of the thoracic aorta are known to correlate with aorta stiffness in older populations. This prospective study aimed to analyze the changes in these FDG-PET/CT features between young, middle-aged, and older adults, and investigate associations with arterial stiffness and blood pressure (BP).

**Methods:**

Young (< 40 years), middle-aged (40-to-60 years), and older (> 60 years) adults, who underwent an FDG-PET/CT, were prospectively recruited. FDG-PET/CT features of the proximal half of the thoracic aorta were analyzed relative to the age categories, BP and carotid-femoral pulse wave velocity (PWV), a reference indicator of aorta stiffness.

**Results:**

We included 79 patients (38 women; 22 young, 19 middle-aged, and 38 older adults). An increase in age category was associated with increases in mean standardized uptake values (SUVs) of blood and aorta and most significantly in aorta SUV heterogeneity, represented by SUV standard deviation (SUV-SD), aorta calcification volume, and the aorta volume indexed to body surface area. However, this indexed aorta volume was the sole variable: (i) exhibiting a stepwise increase from young (median: 25 cm^3^/m^2^ [interquartile range: 20–28 cm^3^/m^2^]), to middle-aged (41 [30–48] cm^3^/m^2^, *p* < 0.001 vs. Young), and older (62 [44–70] cm^3^/m^2^, *p* < 0.001 vs. middle-age) adults, and (ii) selected in the multivariate predictions of systolic, diastolic, and pulse BP. Indexed aorta volume was also a multivariate predictor of PWV but in association with SUV-SD and hypertension.

**Conclusion:**

In a population of patients referred to an FDG-PET/CT investigation, the indexed volume of the proximal and more elastic half of the thoracic aorta is the most comprehensive indicator of arterial aging. This imaging parameter exhibits a stepwise increase from young to middle-aged and older adults, is strongly linked to inter-individual changes in both arterial stiffness and BP, and thus, could help assess the early phases of arterial aging.

*Trial registration* ClinicalTrial.gov, NCT03345290. Registered 17 November 2017, https://clinicaltrials.gov/ct2/show/NCT03345290?term=NCT03345290&draw=2&rank=1

## Background

Aorta function and stiffness are central to the increase in systolic and pulse blood pressure (BP) that occurs with advancing age. Aorta stiffness per se, is a strong indicator of the risk of cardiovascular events (Laurent et al. 2006; Lee et al. 2022) and the gold standard for its noninvasive measurement is the aortic pulse wave velocity (PWV)—i.e., the velocity by which the arterial pulse wave travels along the aorta (Laurent et al. 2006).

Age-related changes in aorta stiffness and function are a multifactorial phenomenon starting in young adulthood and predicated by the extent of: elastin fragmentation, collagen fibrosis, a decrease in the density of muscular smooth cells, inflammation, calcification, and endothelial dysfunction (Raggi et al. 2007; Mackey et al. 2007; Schlatmann et al. 1977; Carlson et al. 1970). Macroscopically, the volume of aorta calcification as well as age-related changes in the length, diameter and distensibility of the thoracic aorta all effect aorta stiffness and PWV (Scott et al. 2020; Craiem et al. 2012; Broyd et al. 2021; Joly et al. 2009a, b; Joly et al. 2016). In healthy adults, the diameter of the thoracic aorta increases on average by about 0.13 cm and its length by about 1.2 cm per decade of life, and results in an increase in aorta volume and an aorta that uncoils with age (Craiem et al. 2012).

Increases in PWV have been shown to be associated with an increase in ^18^F-fluorodeoxy-glucose (FDG) positron emission tomography (PET) uptake in ascending aorta and aortic arch but only in small, selected study populations—i.e., diabetic, hypertensive, and primarily old patients (Joly et al. 2009a, b; Joly et al. 2016; de Boer et al. 2016). In this regard, we know very little about the younger subjects’ population, whereas some may suffer from accelerated arterial aging (Benetos et al. 2002).

This prospective cross-sectional study, planned in a population of patients referred to an FDG-PET/CT investigation, aimed to analyze FDG-PET and CT feature changes in the proximal and more elastic half of the thoracic aorta of young, middle-aged, and older adults, and investigate associations with arterial stiffness and BP.

## Methods

### Study population

We prospectively enrolled 100 participants referred for a FDG-PET/CT to our university hospital between August 2018 and November 2020 for any indication, except treated oncologic disease, as part of the PACTEP study (Central Arterial Pressure and Positron Emission Tomography; Clinicaltrials.gov site number: NCT03345290) (Zimmermann et al. 2022).

Participants included 25 young adults (18-to-40 years), 25 middle-aged adults (41-to-60 years), and 50 older adults (> 60 years) with the latter group providing a larger subgroup of individuals with increased arterial stiffness and BP.

Baseline PWV and brachial BP were measured on the day of the FDG-PET/CT. A baseline PWV was not determined for atrial fibrillation patients.

### Hemodynamic parameters

All the hemodynamic parameters were recorded on the day of the FDG-PET/CT, just before FDG injection. Resting brachial systolic, diastolic BP and heart rate were measured three times with an automatic monitor (DINAMAP v100, General Electric®) and averaged for subsequent analyses. The pulse BP was computed as systolic BP minus diastolic BP.

L.J. and P.S. measured the carotid-femoral PWV by applanation tonometry with the Pulspen DiaTecne® system (San Donato Milanese, Italy) (Salvi et al. 2004; Joly et al. 2009a, b). PWV was obtained by dividing the arterial length estimated by 80% of the distance between the carotid and femoral site, by the time, separating the onset of the pulse waves, recorded on the right common carotid and femoral arteries (Joly et al. 2009a, b; Joly et al. 2016).

### FDG-PET/CT image recording and reconstruction

PET/CT was recorded on a hybrid system with a 64-raw multi-slice CT and a digital-PET camera (Vereos, Philips®, Cleveland, Ohio), starting 60 min after the intravenous injection of 3 MBq/kg of ^18^F-FDG. Participants fasted at least six hours prior to the PET/CT. Unenhanced CT scans were recorded first, from the mid-thigh to the base of the skull using the following parameters: 120 kV, 80 mA with 3D mA modulation according to body weight, and pitch of 0.828. CT slices were reconstructed with an iterative method and displayed as 2 mm thick, 512 × 512 matrices. PET images were subsequently recorded with 8 or 9 consecutive 3-min bed positions and reconstructed with parameters favoring the contrast-to-noise ratio of small structures (Salvadori et al. 2020).

### FDG-PET/CT analysis

PET and CT images were analyzed on a MIM workstation (MIM Software Inc.®, Cleveland, OH, USA). The volume of the first half of the thoracic aorta was segmented on CT slices from an axial plane placed just below the right pulmonary artery to a plane including the bisector between the aortic arch and the descending aorta segments. We used a semi-automatic segmentation method where the external aorta borders were drawn manually on a single CT slice, automatically expanded on the aorta volume and further corrected manually if necessary. This aorta volume of interest (VOI) was used to determine the aorta volume, expressed relative to body surface area (Craiem et al. 2012), as well as the volume of aorta calcification with a conventional 130 Hounsfield Unit threshold (Joly et al. 2009a, b; Joly et al. 2016).

Maximal and mean standardized uptake values (SUVmax and SUVmean, respectively), and the SUV standard deviations (SUV-SD), were extracted from aorta VOIs. Maximal and mean tissue-to-background ratios (TBRmax and TBRmean, respectively) were respectively computed as the ratios of SUVmax and SUVmean over the mean blood SUV obtained from a 2 cm diameter spherical VOI placed at the center of the right atrial cavity (Boursier et al. 2022).

### Statistical analysis

Continuous variables were expressed as medians [with interquartile ranges], and categorical variables as percentages. Unpaired multi-group comparisons between the 3 groups of young, middle-aged, and older subjects were performed with (i) the Chi-square or Fisher’s exact tests for categorical variables and (ii) a non-parametric Kruskal–Wallis test for continuous variables since most variables were not normally distributed. Associations between continuous variables were tested with the non-parametric Spearman’s rank correlation method. A p-value < 0.05 was considered to be significant.

Two-group comparisons obtained from the 3 age-groups were performed using the Mann–Whitney test for continuous variables, Chi-square or Fisher’s exact tests for categorical variables, and with a *p*-value < 0.025 considered to be significant to account for test multiplicity.

Multivariate linear regression models were used to analyze associations between individual hemodynamic parameters—i.e., PWV, systolic, diastolic, and pulse BP—and the clinical and PET/CT variables listed in Tables 1 and 2. Variables with *p* < 0.15 from the univariate analysis were initially entered and a final model was selected using a stepwise backward approach which only retained variables with *p* < 0.05. Regression model fit was checked for distribution of residuals and with homoscedasticity tests. Linearity between outcome and continuous variables was ascertained using restricted cubic spline analyses.

**Table 1:** Main clinical and hemodynamic data of the overall population with the corresponding age-stratified comparisons between young (≤ 40 years old), middle-aged (41 to 60 years old), and older (> 60 years old) adults

	Overall (*n* = 79)	Young (*n* = 22)	Middle-aged (*n* = 19)	Old (*n* = 38)	*P*-value
Women	38 (48%)	11 (50%)	13 (68%)	14 (37%)	0.080
Age (years)	59 [38–70]	31 [27–38]	55 [48–57]*	71 [65–75]*^†^	< 0.001
BMI (kg/m^2^)	27.2 [23.4–30.5]	25.2 [22.1–29.0]	29.1 [26.9–32.7]	27.3 [23.9–29.3]	0.129
Obesity	22 (28%)	5 (23%)	8 (42%)	9 (24%)	0.316
Hypertension	35 (43%)	3 (14%)	5 (26%)	27 (71%)*^†^	< 0.001
Diabetes	7 (9%)	1 (5%)	0 (0%)	6 (16%)	0.169
Dyslipidemia	33 (42%)	2 (9%)	6 (32%)	25 (66%)*^†^	< 0.001
Smoking	20 (25%)	9 (41%)	3 (16%)	8 (21%)	0.128
History of CV disease	11 (14%)	0 (0%)	2 (11%)	9 (24%)	0.022
Heart rate (bpm)	64 [56–73]	61.5 [55–72]	68 [56–79]	63.5 [57–70]	0.615
Systolic BP (mmHg)	125 [116–137]	117 [112–122]	127 [119–137]*	132 [121–147]*	< 0.001
Diastolic BP (mmHg)	71 [64–79]	66 [63–72]	74 [67–77]	72 [64–83]	0.147
Pulse BP (mmHg)	54 [48–64]	50 [43–53]	56 [48–64]*	58 [53–76]*	0.002
PWV (m/s)	7.5 [6.1–9.7]	6.3 [5.9–6.7]	7.2 [6.7–9.3]*	8.6 [7.3–11.5]*	0.001

Continuous variables expressed as medians [with interquartile ranges] and categorical variables as n (%)

BMI: body mass index; bpm: beats per minute; CV: cardiovascular; BP: blood pressure; PWV: Pulse wave velocity

^*^significant difference vs. young adults; ^**†**^significant difference vs. middle-aged adults

**Table 2:** Main PET/CT data extracted from the analysis of blood and the proximal half of thoracic aorta of the overall population, with the corresponding age-stratified comparisons between young (≤ 40 years old), middle-aged (41 to 60 years old), and older (> 60 years old) adults

	Overall (*n* = 79)	Young (*n* = 22)	Middle-aged (*n* = 19)	Old (*n* = 38)	*P*-value
Blood SUVmean	1.78 [1.53–2.07]	1.54 [1.41–1.71]	1.85 [1.54–2.10] *	1.85 [1.68–2.11]*	0.002
Aorta volume (cm^3^/m^2^)	43 [28–62]	25 [20–28]	41 [30–48] *	62 [44–70]*†	< 0.001
Aorta calcification volume (cm^3^)	0.05 [0.00–0.81]	0.00 [0.00–0.00]	0.00 [0.00–0.12]	0.81 [0.27–2.19]*†	< 0.001
Aorta SUVmax	2.98 [2.54–3.35]	2.61 [2.43–3.26]	2.98 [2.54–3.36]	3.07 [2.80–3.57]	0.080
Aorta SUVmean	1.84 [1.64–2.04]	1.68 [1.47–1.83]	1.90 [1.64–2.12]	1.88 [1.72–1.99]*	0.041
Aorta SUV-SD	0.26 [0.23–0.30]	0.22 [0.19–0.24]	0.26 [0.23–0.28]	0.27 [0.25–0.34]*	< 0.001
Aorta TBRmax	1.62 [1.52–1.82]	1.65 [1.60–1.77]	1.62 [1.55–1.73]	1.57 [1.48–1.86]	0.296
Aorta TBRmean	1.03 [0.98–1.09]	1.09 [1.05–1.13]	1.05 [0.99–1.13]	1.00 [0.94–1.05]*	< 0.001

Continuous variables expressed as medians [with interquartile ranges] and categorical variables as *n* (%)

SD: standard deviation; SUV: standardized uptake value; TBR: tissue-to-background ratio

^*^significant difference vs. young adults; ^**†**^significant difference vs. middle-aged adults

## Results

### Participant characteristics

We initially enrolled 100 participants with 21 participants subsequently excluded because of incomplete or unavailable data, known or suspected vasculitis, or tumor or adenopathy in close contact with the aorta. The remaining 79 participants: 38 women, 22 young adults (< 40 years), 19 middle-aged (40-to-60 years), and 38 older adults (> 60 years), were finally included in the analyses. We respectively obtained brachial BP and accurate PWV measurements from all and 68 participants. The main reasons for referral to FDG-PET were: an initial diagnostic workup for cancer (*n* = 16), known granulomatosis (*n* = 17) or other inflammatory diseases (*n* = 16), adenopathy of unknown origin (*n* = 11), infectious diseases (*n* = 6), and others (*n* = 13). None had any sign of vasculitis.

Stratification into young, middle-aged and older adult groups identified age-related increases in the BP level and PWV, as well as increased rates of medically-treated hypertension and dyslipidemia, with higher values observed in older compared to younger adults for these clinical and hemodynamic variables examined (Table 1). Significant increases in BP and PWV levels were already observed between the young and middle-aged groups, whereas the rates of antihypertensive treatment increased later, in the older patient group (Table 1).

### Comparison of FDG-PET/CT data between young, middle-aged, and older adults

**Fig. 1:**
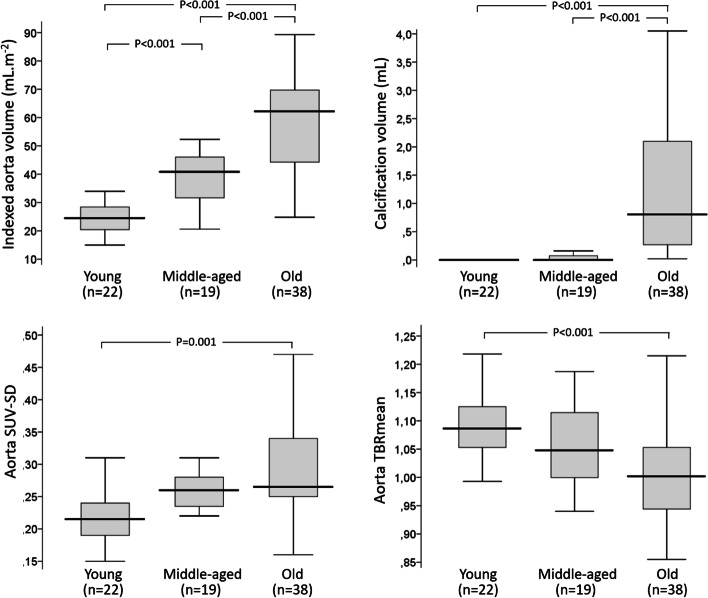
Comparisons between the three age-groups (i.e., young (< 40 years), middle-aged (40-to-60 years), and older (> 60 years) adults) in terms of aorta volume and aorta calcification determined from CT images (upper panels), and of the aorta SUV standard deviation (SUV-SD) and aorta-to-blood SUV mean ratio (TBRmean) extracted from FDG-PET images (lower panels)

As detailed in Table 2 and Fig. 1, the aorta calcification volume extracted from CT images was a strong predictor of age, with significantly higher aorta calcification volumes observed in older than in both young and middle-aged adults. However, the indexed aorta volume exhibited a clear stepwise increase between the young (median: 25 cm^3^/m^2^ [interquartile range: 20–28 cm^3^/m^2^]), middle-aged (41 [30–48] cm^3^/m^2^, *p* < 0.001 vs. Young), and older (62 [44–70] cm^3^/m^2^, *p* < 0.001 vs. middle-age) adult groups (Table 2, Fig. 1).

An increase in age category also correlated with an increase in SUVmean from both blood and aorta, with the highest values for both variables observed in the older age-group (Table 2). The most significant age-related differences between FDG-PET variables were: (i) the aorta TBRmean that progressively decreased from younger to older adults (*p* < 0.001, Fig. 1), and (ii) the aorta SUV-SD, an index of SUV heterogeneity that progressively increased from younger to older adults (*p* = 0.001, see Fig. 1).

**Fig. 2:**
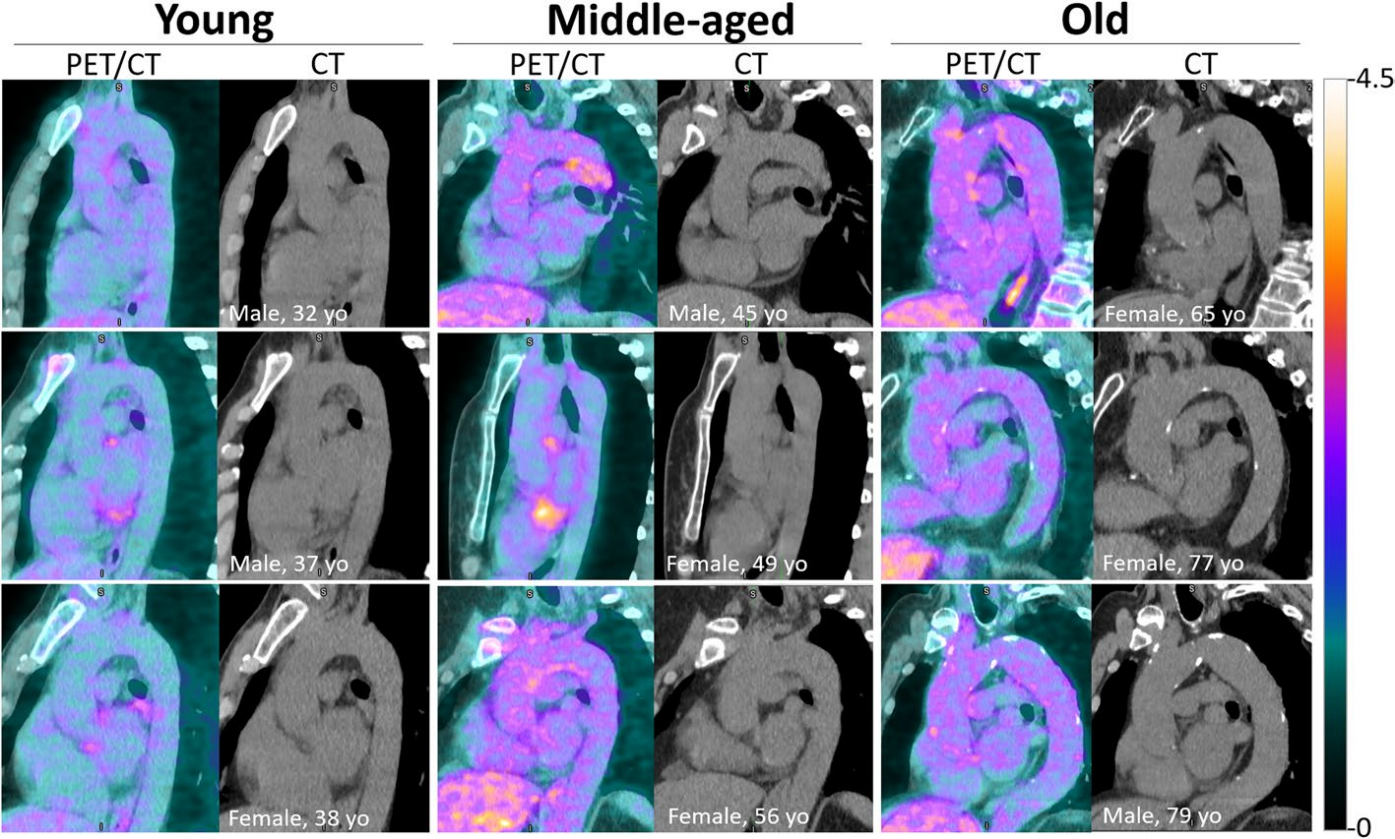
Representative paired fused PET/CT and CT aorta plane slices of young, middle-aged, and older adults (3 individuals per age-group). The color scale legend for PET images is shown on the right. Aorta calcifications are only observed on CT slices of the older age-group. Note the changes in intensity and heterogeneity of aorta FDG activity and aorta volume with the aorta uncoiling with increasing age

Representative PET and CT slices from each age-group are shown in Fig. 2.

### Correlates of PWV and BP levels

**Fig. 3:**
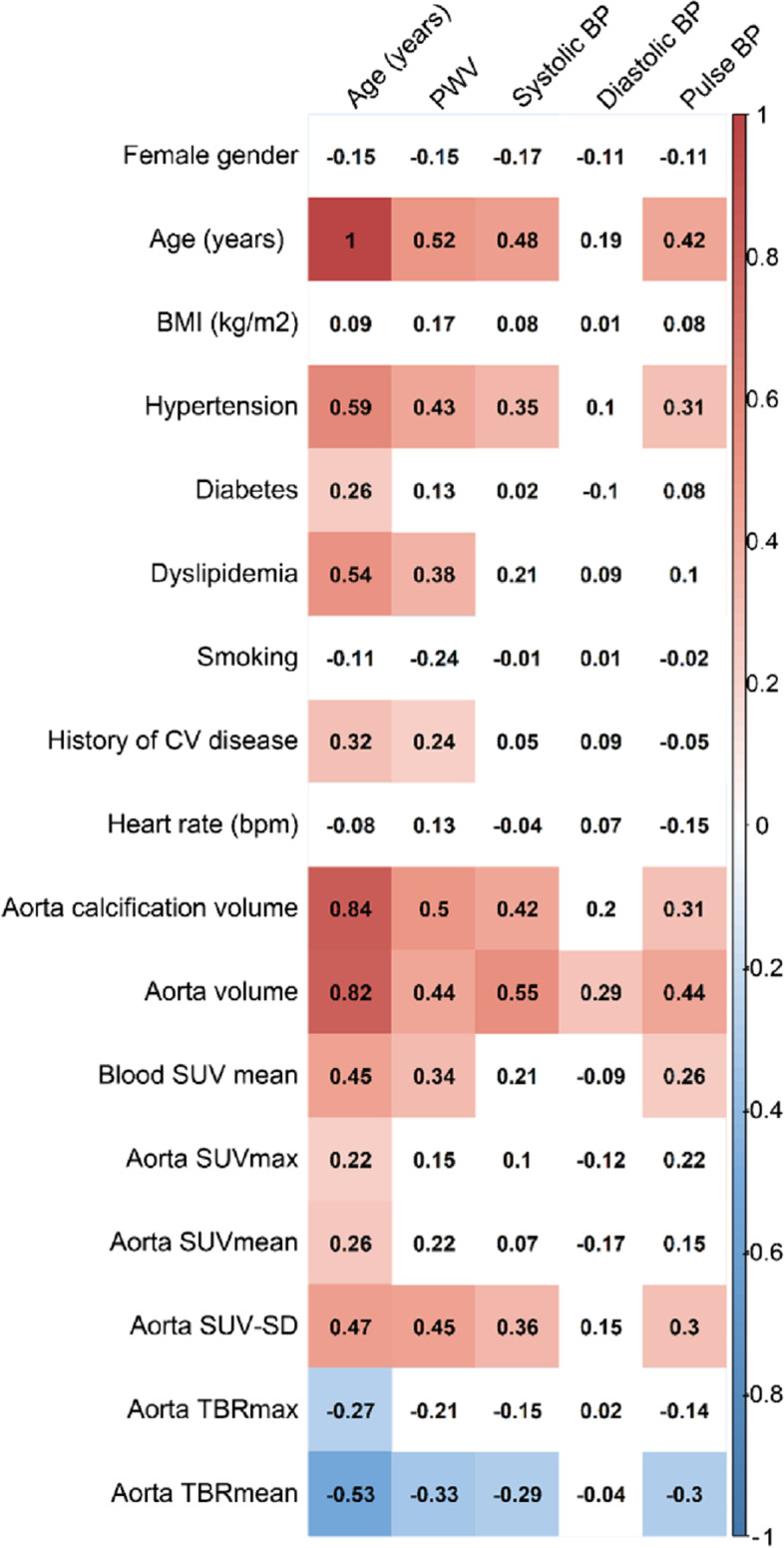
Comparisons of the Spearman correlation coefficients (rs Spear.) of the (i) clinical and PET/CT data and (ii) age and hemodynamic parameters (PWV, systolic, diastolic, and pulse BP)

As detailed in Fig. 3, indexed aorta volume was the sole significant univariate correlate of diastolic BP (Spearman coefficient of 0.29, *p* = 0.01,) and this variable was also strongly correlated with all other hemodynamic variables (PWV, systolic BP, and pulse BP), with Spearman coefficients ranging from 0.44 to 0.55. The other strong correlates of hemodynamic variables (i.e., Spearman correlation coefficients ≥ 0.3) were calcification volume, but also TBRmean, SUV-SD, blood SUV, hypertension and age (Fig. 3).

In the multivariate analysis, all aorta PET/CT variables and heart rates should be dichotomized according to the median, and PWV should be log-transformed. As detailed in Table 3, the criteria of an indexed aorta volume exceeding the median value was always selected in the final models built to predict ln(PWV), systolic, diastolic, or pulse BP. Furthermore, this aorta volume parameter was the sole multivariate predictor selected, with the exception of the ln(PWV) prediction, where two additional variables were kept in the final model: hypertension and SUV-SD dichotomized according to the median.

**Table 3:** Independent multivariate predictors of ln(PWV), systolic BP, diastolic BP, or pulse BP, among all considered clinical, PET, and CT variables

	Ln(PWV)		Systolic BP		Diastolic BP		Pulse BP	
	Beta ± SE	*P*-value	Beta ± SE	*P*-value	Beta ± SE	*P*-value	Beta ± SE	*P*-value
Hypertension	0.163 ± 0.077	0.037						
Aorta SUV-SD > median	0.145 ± 0.070	0.041						
Aorta volume > median	0.169 ± 0.075	0.027	19.459 ± 3.819	< .0001	13.661 ± 3.026	< .0001	13.661 ± 3.026	< .0001

SD: standard deviation; SUV: standardized uptake value

When age was forced into the models, the criteria of an indexed aorta volume exceeding the median value still provided a significant additional predictor for systolic BP (*p* = 0.004), but not for PWV (*p* = 0.24), diastolic BP (*p* = 0.34) or pulse BP (*p* = 0.11).

## Discussion

This current prospective study shows that the indexed volume of the proximal and more elastic half of the thoracic aorta (i.e., ascending aorta and aortic arch) is a strong indicator of arterial aging. We provide supportive evidence of a clear stepwise increase in this indexed volume from young to middle-aged, and older adults, and identify this volume parameter as the most comprehensive FDG-PET/CT predictor of the inter-individual changes in both arterial stiffness and BP.

We examined several hemodynamic variables, namely (i) PWV, a non-invasive gold standard for assessing aorta stiffness (Laurent et al. 2006), (ii) systolic and pulse brachial BPs, which are strongly impacted by the stiffness of the great vessels (Schiffrin et al. 2004), and (iii) diastolic BP which likely sustains blood flow through the microvasculature (McEvoy et al. 2016). The stiffness-related hemodynamic variables (i.e., PWV, systolic BP, and pulse BP) were strongly related to the age stratification, with significant increases identified between the young and middle-aged population, whereas the rates of treated hypertension increasing in later life (i.e., between the middle-aged and older patient group) (see Table 1). The early occurrence of hemodynamic changes supports the common consideration that arterial stiffening and associated histologic changes start in young adulthood (Schlatmann et al. 1977; Díaz et al. 2014; Reference Values for Arterial Stiffness’ Collaboration. 2010), but contrasts with the widespread and significant FDG-PET and CT variable changes mainly observed in our older subjects (Table 2).

Among imaging variables, indeed, only the indexed aorta volume exhibited a significant increase from young to middle-aged adults, with a further increase from middle-aged to older adults (Fig. 1). The median aorta volume was 64% higher in the middle-aged group and 248% higher in the older group, when compared to the younger age-group (Table 2).

**Fig. 4:**
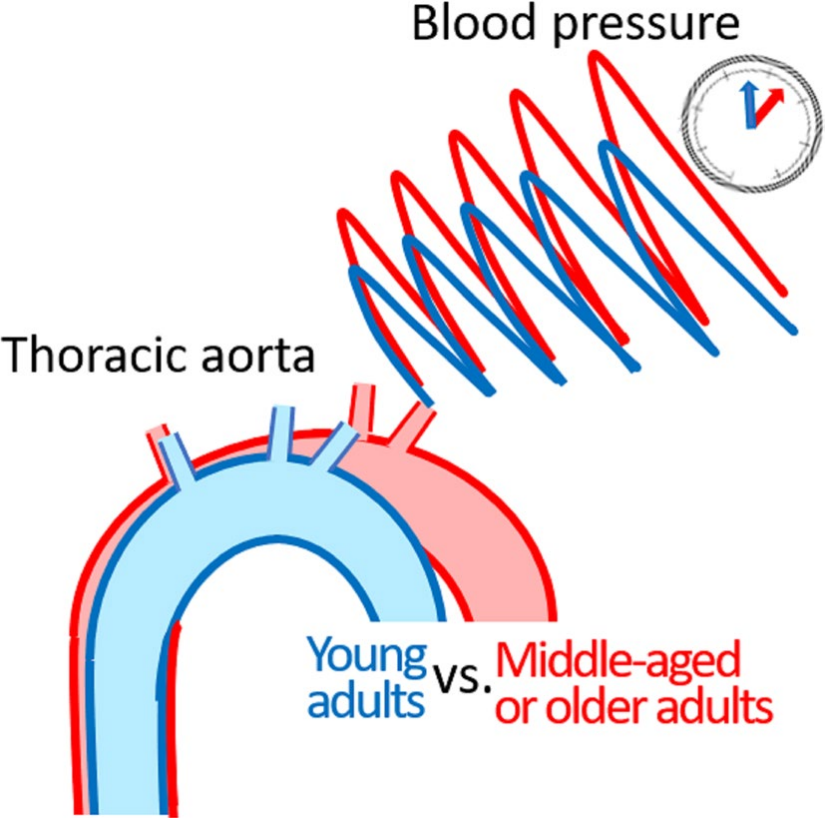
Schematic representation of the concordant increases in aorta volume and blood pressure observed in middle-aged or older adults as compared to young adults

A schematic representation of the concordant increases in aorta volume and blood pressure, which are observed in middle-aged or older adults, as compared to young adults, is displayed in Fig. 4.

We further identified, indexed aorta volume to be an independent multivariate predictor for each of the arterial hemodynamic variables examined (i.e., PWV, systolic, diastolic, and pulse BP), contrary to FDG-PET and clinical variables, and to outperform the prediction provided by age for systolic BP, a key indicator of cardiovascular risk (Flint et al. 2019). This strong association between indexed aorta volume and systolic-BP may be explained by the wall stress variations induced by systolic BP which impacts the lumen of the thoracic aorta in the short term (i.e., the systolic BP-aorta diameter association) (Stefanadis et al. 1995; Toutouzas et al. 2000), but also in the longer term, as previously shown in populations of patients with hypertension (Cesareo et al. 2021; Wang et al. 2022; Tadic et al. 2022) or various cardiovascular risk factors (Chen et al 2022), and Marfan syndrome (Brooke et al. 2008).

The association of aorta volume with stiffness is in line with the previously published report of correlations between aorta diameter and PWV (Bailey et al. 2014), as well as between aorta size and distensibility (i.e., the increase in an ascending aorta 2D size between diastole and systole) (Broyd et al. 2021). However, the changes in aorta volume, which were computed here, are likely to better reflect the 3D effects of age, as compared with changes in aorta diameter, length and other 1D or 2D parameter.

All together the data substantiates indexed aorta volume, measured in the proximal and more elastic half of the thoracic aorta, as a strong indicator of arterial aging throughout adulthood.

We also found aorta calcification of the proximal half of the thoracic aorta to be highly correlated with age and hemodynamic parameters. The significant increase in calcification volume was however only apparent in the older age-group and not in middle-aged participants, contrary to what was observed for aorta volume (Fig. 1). The low rates of diabetes in our young and middle-aged groups (only one patient) could also partly explain this last observation, with diabetes being the most potent risk factor for early arteriosclerotic calcification (Tsai et al. 2019). Results provided by PET with ^18^F-sodium fluoride could also be of interest in this setting, given that this tracer provides detection of microcalcifications, which are yet invisible on CT (Reijrink et al. 2022).

Among FDG-PET variables, the SUVmean of the first half of the thoracic aorta was significantly related to the age-based stratification. An even stronger association was observed for blood SUVmean (Table 2). This age-dependence of the blood level of FDG activity is consistent with results from previous studies (Cao et al. 2021), and presumably reflects the emergence of insulin resistance during the aging process (Barzilai et al. 2012).

We were however unable to confirm the association between aorta SUV and PWV, which was previously reported in very specific populations of old subjects (Joly et al. 2009a, b; Joly et al. 2016). In the present large age-stratified FDG-PET/CT population, a substantial correlate of age and hemodynamic variables was observed in terms of the standard deviation of aorta SUV (SUV-SD), an index of SUV heterogeneity that increased with age (Figs. 1 and 3). This aorta SUV heterogeneity likely relates to the coexistence of areas with high FDG uptake, due to the presence of atherosclerotic lesions (Small et al. 2011; Fernández-Friera et al. 2019), and low uptake areas which may reflect the decrease in smooth muscle cell density with increasing age (Schlatmann et al. 1977; Carlson et al. 1970).

### Main limits

The limited number of patients, especially in the young and middle-aged adult groups, constitutes a first limitation. In addition, the observed relationship between the indexed volume of the proximal half of the thoracic aorta and functional signs of arterial aging (BP, PWV) needs to be confirmed at a larger scale, by longitudinal studies, and moreover, in other study populations than the present one—i.e., in patients without any suspected health disease. It is of note that some patients had a known or suspected inflammatory or infectious disease, but none had any known or suspected vasculitis.

## Conclusion

In a population of patients referred to an FDG-PET/CT investigation, the age-stratification into young, middle-aged, and older adult subgroups, and the analysis of associated changes in BP and arterial stiffness, identified a strong association with proximal aorta volume, which corresponds to the more elastic half of the thoracic aorta (i.e., ascending aorta and aortic arch). This imaging parameter, which is very easy to record and analyze, is the most comprehensive FDG-PET/CT indicator of arterial aging during adulthood, including in young and middle-aged adults, and it could potentially help assessing and further preventing accelerated arterial aging, especially during the early phases.

## Data Availability

The datasets used and/or analysed during the current study are available from the corresponding author on reasonable request.

## Abbreviations

BP: Blood pressure
CT: Computed tomography
FDG: ^18^F-Fluorodesoxyglucose
LV: Left ventricle
PET: Positron emission tomography
PWV: Aortic pulse wave velocity
SD: Standard deviation
SUV: Standardized uptake value
TBR: Tissue-to-background ratio
VOI: Volume of interest
